# Spectral Analysis of Acceleration Data for Detection of Generalized Tonic-Clonic Seizures

**DOI:** 10.3390/s17030481

**Published:** 2017-02-28

**Authors:** Hyo Sung Joo, Su-Hyun Han, Jongshill Lee, Dong Pyo Jang, Joong Koo Kang, Jihwan Woo

**Affiliations:** 1 Department of Biomedical Engineering, University of Ulsan, Ulsan 44610, Korea; mywngprud1@gmail.com; 2 Department of Neurology, Chung-Ang University College of Medicine, Seoul 06973, Korea; freesu84@naver.com; 3 Department of Biomedical Engineering, Hanyang University, Seoul 04763, Korea; netlee@bme.hanyang.ac.kr (J.L.); dongpjang@gmail.com (D.P.J.); 4 LGT Neuro Medical Center, Seoul 06106, Korea; jkkang@amc.seoul.kr

**Keywords:** epilepsy, seizure detection, accelerometer, spectral analysis

## Abstract

Generalized tonic-clonic seizures (GTCSs) can be underestimated and can also increase mortality rates. The monitoring devices used to detect GTCS events in daily life are very helpful for early intervention and precise estimation of seizure events. Several studies have introduced methods for GTCS detection using an accelerometer (ACM), electromyography, or electroencephalography. However, these studies need to be improved with respect to accuracy and user convenience. This study proposes the use of an ACM banded to the wrist and spectral analysis of ACM data to detect GTCS in daily life. The spectral weight function dependent on GTCS was used to compute a GTCS-correlated score that can effectively discriminate between GTCS and normal movement. Compared to the performance of the previous temporal method, which used a standard deviation method, the spectral analysis method resulted in better sensitivity and fewer false positive alerts. Finally, the spectral analysis method can be implemented in a GTCS monitoring device using an ACM and can provide early alerts to caregivers to prevent risks associated with GTCS.

## 1. Introduction

There are concerns that unwitnessed seizures might cause injury or even death in people with epilepsy, because most patients and families cannot always predict when a seizure might occur [1]. The mortality rate is known to increase if generalized tonic-clonic seizures (GTCSs) are involved [2,3]. Supervision by sharing a room can reduce the risks related to GTCS as the supervisor can render assistance by repositioning, removing a respiratory obstruction, or stimulation [4]. In addition concerns about the possible complications of GTCSs and the number of GTC events are sometimes underestimated due to unconsciousness and confusion [5]. The underestimation of GTC events may also cause a delay in optimal management and an increase in complications of GTCS. Thus, methods for detecting potentially dangerous seizures, especially GTCS, are essential and useful for early intervention during events by caregivers and for the exact estimation of the events [1].

Electroencephalography (EEG), accelerometry (ACM), electromyography, and sympathetic mediated electrodermal activity have been developed to detect seizure events [1,5,6,7,8,9,10,11]. EEG is the gold standard for seizure diagnosis. Many research groups have investigated EEG-based detection systems [9,12,13]. Although EEG-based systems provide comparably high sensitivity, EEG-based systems have limitations due to the inconvenience of requiring an electrode to be placed on the scalp during daily life.

Since GTCSs include repetitive and jerking movements and stiffening of the muscles, these repetitive motions occurring during GTCS can be used as key features for detecting GTCSs. Some studies have suggested that methods using ACM can detect such movements, are more comfortable, and can detect seizure-related motion with fewer restrictions than EEG-based measures do [7,10]. Several detection algorithms have been studied using ACM data processing [1,5,8,11,14]. Various temporal features in ACM data, such as standard deviation, jerk, and delta-jerk, have been used to quantify GTC events [5,7,15]. In addition, several studies have investigated seizure motion classification with spectral information via short-time Fourier transform, spectral density computation, and/or wavelet transform [10,16]. However, developing more accurate and reliable algorithms for GTCS detection with higher sensitivity and lower false positive predictive values still remains a challenge.

The aim of the present study was to develop a novel algorithm on the basis of the spectral analysis of ACM data. The seizure-dependent weighting feature was calculated based on the spectral information of the ictal data and was used to detect GTCSs. The spectral analysis method was evaluated and compared to temporal analysis methods in terms of sensitivity and the false positive rate.

## 2. Materials and Methods

### 2.1. The Wireless Sensor

**Figure 1:**
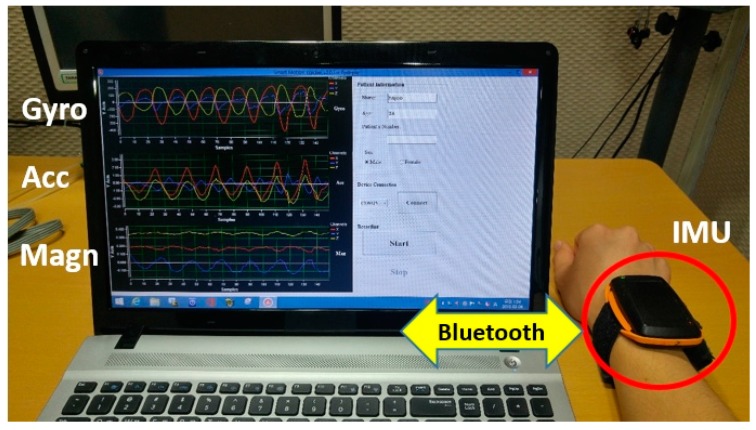
The experimental setup with the inertia measurement unit (IMU). The IMU is placed on the patient’s wrist and sends motion data to a laptop computer via Bluetooth.

The inertial measurement unit (IMU) was developed to detect the arm motion of patients, as seen in Figure 1. The band-type IMU used in this study consisted of an MSP430 microcontroller (MSP430F1611, Texas Instruments, San Diego, CA, USA) and low-cost inertial sensors of a three-axis accelerometer and magnetometer (LSM303DLM, STMicroelectronics, Geneva, Switzerland). A rechargeable battery (PSE H503438-PCM (3.7 V, 680 mAh), PSE, Jinan, China) was embedded and guaranteed approximately 8 h of operation. The IMU was designed to sense ±8 g acceleration and the sampling ratio was set at 100 samples/s. The sensing data parameters were preprocessed in the microcontroller and transmitted via the embedded Bluetooth module (Parani-ESD200, Sena Technologies Inc., Seoul, Korea) to a portable computer. The data acquisition was controlled by a custom-made PC program. In this study, acceleration data were used to measure patient motion and the vector magnitude of the three-axis accelerations (*x*[*n*], *y*[*n*], and *z*[*n*]) were computed as ${\left(x{\left[n\right]}^{2}+y{\left[n\right]}^{2}+z{\left[n\right]}^{2}\right)}^{1/2}$ and used as ACM data.

### 2.2. Subjects

**Figure 2:**
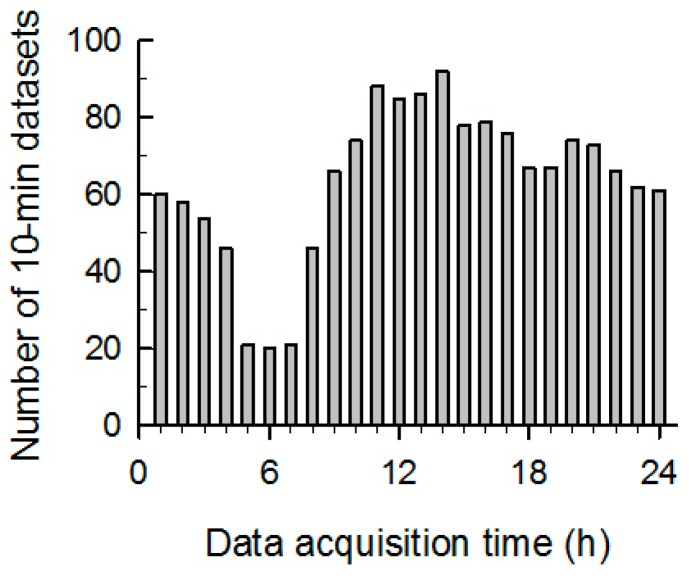
Distribution of acceleration data acquisition times from 12 patients. The vertical axis represents the number of 10-min datasets recorded during an hour interval.

Twelve epileptic patients who had undergone scalp video-EEG monitoring with the 10-10 international system including sphenoidal electrodes for pre-surgical work-up participated in this study. They were 13 to 52 years old (seven males and five females). Patient information is shown in Table 1. During the video-EEG monitoring, the IMUs were worn on the wrists of the patients to monitor forearm motion. Batteries were replaced with fully-recharged ones every 8 h. Among the 12 patients, five patients had a total of 10 GTCSs and the other seven patients did not have a GTCS. The total recording duration of ACM data from all subjects was 246 h, which included GTCS incidents and normal movements (defined as all movements in daily life, including sleep, except those that occur during a GTCS). The video-EEG monitoring data including clinical information, seizure recording times, and the ictal events including the duration of GTCS are listed in Table 1. The seizure intervals were confirmed by the clinician and electroencephalographers based on video-EEG monitoring. As seen in Figure 2, the data collection times were distributed across a whole day, i.e., included both day and night times. Recording time indicates the total duration for data collection, including both ictal and interictal states. GTCS duration represents how long each GTCS lasted after the onset of the GTCS. This study was reviewed and approved by the Asan Medical Center Institutional Review Board (No. 2014-1039).

### 2.3. Spectral Analysis Method for ACM Data

The GTCS ACM data, *x_GTCS_*[*n*], segmented from the dataset, were used to acquire GTCS-dependent spectral information. The frequency components of *x_GTCS_*[*n*] were calculated by fast Fourier transform as follows:

(1) $$X_{GTCS}{\left[\omega \right]}_{m,i}=\sum_{n=0}^{N-1}x_{GTCS}[n+mN]\cdot e^{-j\omega n}$$

where *i* is the number of a GTCS dataset and m is the number of windows for real-time processing, for which the total size, *N*, was set to 100 samples (equivalent to a 1-s duration). The 1-s Hamming window with 50% overlap was used. The spectrum information of GTCS segments (*m*) was averaged and normalized by

(2) $$X_{GTCS}{\left[\omega \right]}_{i}=\frac{\sum_{m=1}^{M}X_{GTCS}{\left[\omega \right]}_{m,i}}{\sum_{\omega =0}^{f_{\max}}\sum_{m=1}^{M}X_{GTCS}{\left[\omega \right]}_{m,i}}$$

where *m* is the number of segments and *f_max_* was set to 50 Hz. The template of averaged GTCS-dependent spectral information, *X_GTCS_*[*ω*], was calculated by averaging nine *X_GTCS_*[*ω*]*_i_* computed from each nine-parameter seizure data point as follows:

(3) $${\bar{X}}_{GTCS}[\omega ]=\frac{1}{N_{s}}\sum_{i=1}^{N_{s}}X_{GTCS}{\left[\omega \right]}_{i}$$

The averaged spectrum information of non-GTCS ACM data (i.e., normal movement), *X_interictal_*[*ω*], was computed similarly to the *X_GTCS_*[*ω*] calculations. Finally, the GTCS-dependent spectral weight function (GDF) was calculated by

(4) $$GDF[\omega ]=\frac{{\bar{X}}_{GTCS}[\omega ]}{{\bar{X}}_{\mathrm{int}erictal}[\omega ]}$$

**Figure 3:**
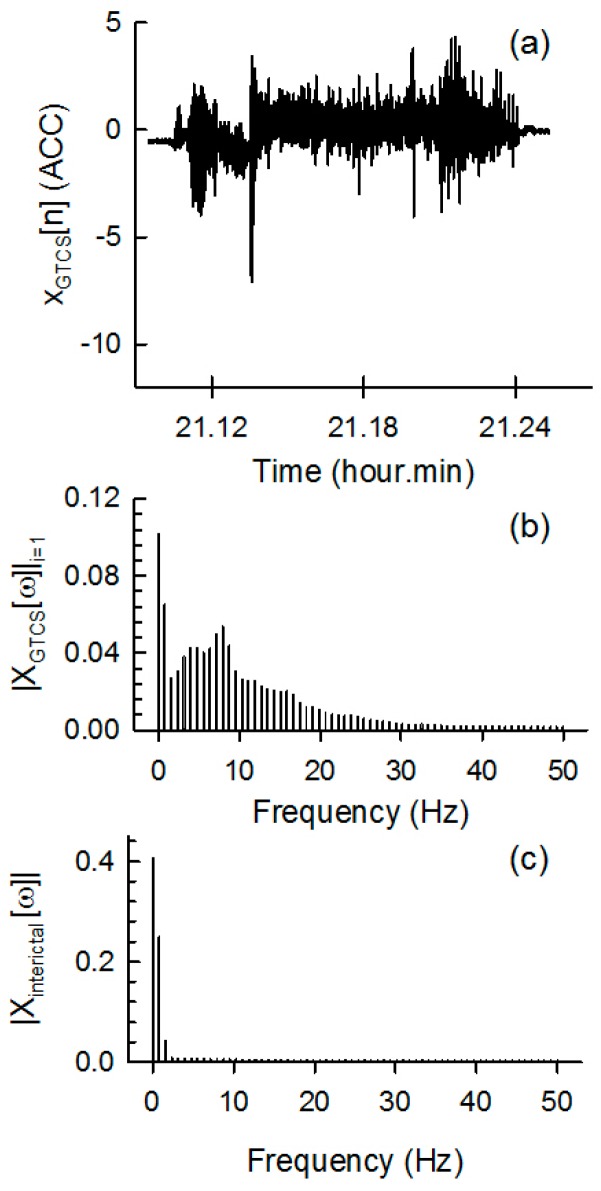
Example of (**a**) the GTCS ACM data as a function of recording time; (**b**) the spectral information of (**a**); and (**c**) the spectral information of the non-GTCS ACM data (interictal).

An example of ACM data for a typical seizure interval (*X_GTCS_*[*n*]), the spectral information (*X_GTC_*_S_[*ω*]) of seizure interval data, and the spectral information (*X_interictal_*[*ω*]) of an interictal interval from the 222 h of interictal data are presented in Figure 3a–c, respectively. Dominant spectral components between 4 and 25 Hz were observed for the GTCS ACM data, whereas only spectral information below 4 Hz was observed for the interictal data.

**Figure 4:**
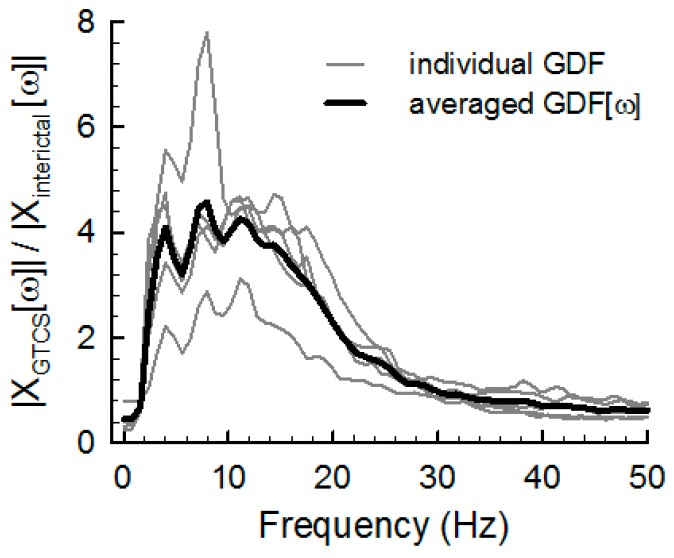
Example of the individual GTCS-dependent spectral weight functions (GDFs).

The GDF represents the spectral power ratio of GTCS to interictal data at each frequency from 0 to 50 Hz. This implies that the GDF describes how GTCSs impact each spectral component relative to non-GTCS movement. For example, if the GDF is 1 at ω = ω_1_, both GTCS movement and non-GTCS movement produce identical power at frequency ω_1_; if the GDF is above 1 at ω = ω_1_, GTCS movement is more correlated to the ω_1_ spectral component than non-GTCS movement is. The GDF was used to compute a weighted average of the spectral powers of ongoing ACC data to detect the GTCS state. Figure 4 shows individual GDFs and the averaged GDF from the 10 individual GDFs. The averaged GDF clearly represents a higher spectral power between 4 and 25 Hz.

A 1-s *m*-th subset (*X_real_*[*n*]*_m_*) of the ACM data and spectral information (*X_real_*[*ω*]*_m_*) were used to detect GTCS motion in real time. The next subset, *X_real_*[*n*]*_m+1_*, was sampled from the next 1-s sampled data set with a 50% overlap with the previous subset, *X_real_*[*n*]*_m_*. The seizure-correlated ratio (SCR) for each *m*-th subset was calculated as follows:

(5) $$SCR[m]=\frac{\sum_{\omega =0}^{f_{\max}}GDF[\omega ]\cdot X_{real}{\left[\omega \right]}_{m}}{\sum_{\omega =0}^{f_{\max}}X_{real}{\left[\omega \right]}_{m}}$$

where *f_max_* was set to 50 Hz due to the 100 Hz sampling rate of data acquisition. Therefore, *SCR*[*m*] was discretely calculated with an interval of 0.5 s. The average of 10 successive SCRs (equivalent to a 5-s average) was compared to a pre-defined threshold to detect a GTC event. If an SCR is higher than the threshold, a GTCS movement has occurred.

A previous study [5] used the parameter of the standard deviation (*Stdev*) of ACM data to detect a GTCS. The repeated shaking movements accompanied by a GTCS can produce a high temporal variation in ACM data and result by increasing a *Stdev*. The *Stdevs* of the *m*th subset of *X_real_*[*n*]*_m_* were calculated as follows:

(6) $$Stdev[m]={\left\{\frac{1}{N}\sum_{n=0}^{N-1}{\left(x_{real}{\left[n\right]}_{m}-M[m]\right)}^{2}\right\}}^{\frac{1}{2}}$$

where the number of subsets of *N* was set at 500 (equivalent to a 5-s duration).

### 2.4. Evaluation of Spectral and Temporal Analyses

We employed 10-fold cross-validation to evaluate the performance of the spectral analysis method using a total of 246 h of ACM data. ACM data included 10 ictal data (GTCSs) and 246 h of interictal data (normal movements) that was divided into 10 data subsets. Each dataset consisted of one ictal event (GTCS) and interictal states, for which durations ranged from 89 s to 256 s and 24 h, respectively. Among the 10 datasets, nine datasets (called the ‘training’ datasets) were used to develop the GDF and one dataset (called the ‘test’ dataset) was tested to evaluate the GTCS detection algorithm. Finally, the 10 results from the 10-fold cases were averaged to compare the performances of the GTCS detection algorithms. The sensitivity, false predictive rate per day (FPR), specificity, and positive predictive value (PPV) for temporal analysis and spectral analysis were computed. Sensitivity represents the ratio of detected seizures to total seizures; FPR, the number of falsely detected seizures per day; specificity, the ratio of normal movement events that are correctly identified to all normal movement events; and PPV, the ratio of correctly detected seizures to total detected movements.

## 3. Results

### 3.1. GTCS Detection

**Figure 5:**
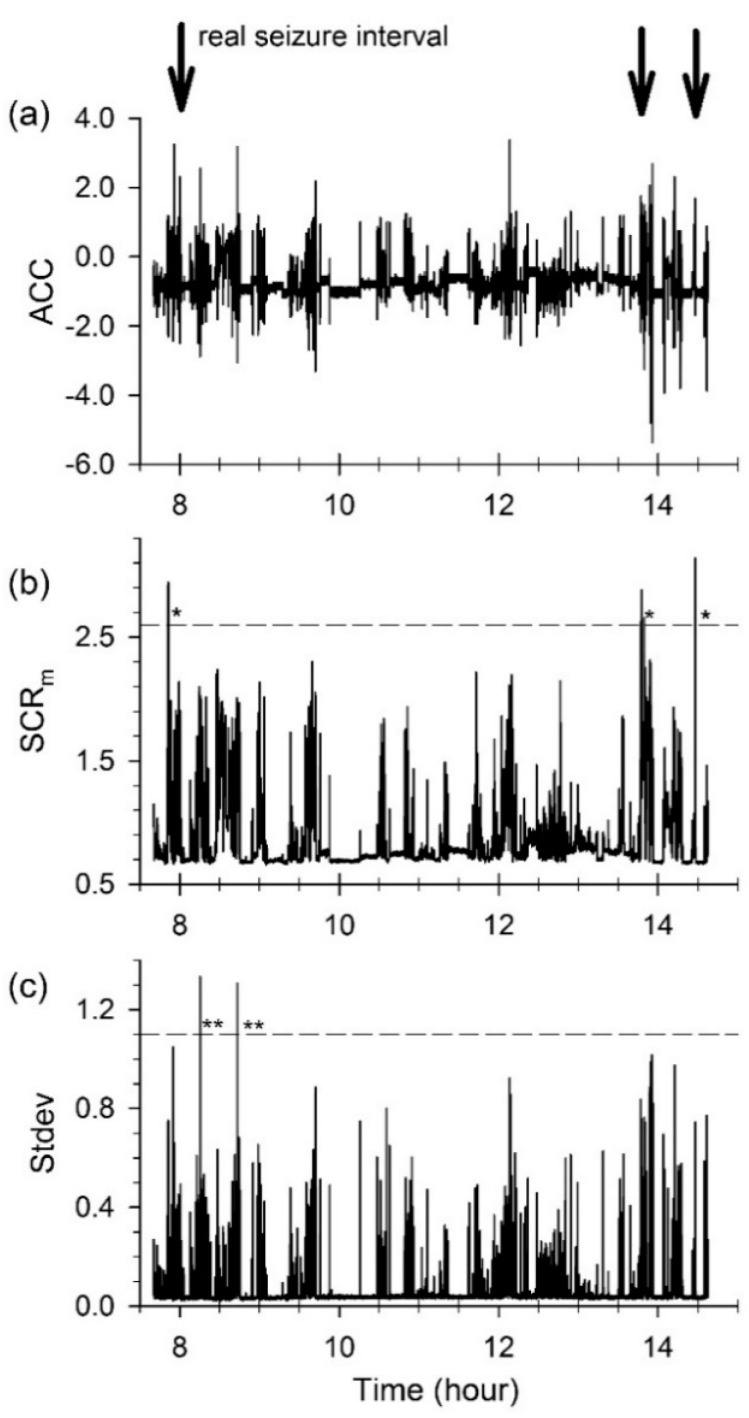
Example of (**a**) acceleration data recorded from 07:30 a.m. to 2:40 p.m.; (**b**) the seizure correlation ratio (SCR) calculated by the spectral analysis method; and (**c**) the standard deviation of the acceleration data of (**a**). ‘*’ and ‘**’ denote true positives and false positives, respectively. See the text for more details.

An actual example of seizure detection is shown in Figure 5. In Figure 5a, the ACM data recorded over 8 h (from 07:30 a.m. to 2:40 p.m.) is plotted versus the recording time, and the ictal intervals are marked by three arrows. Apart from the marked ictal intervals, there were several seizure-like interictal intervals, where high-amplitude variations were observed. The SCR generated by the spectral analysis method and the Stdev calculated by the temporal analysis method are plotted in Figure 5b,c, respectively. In the SCR plot, three high-peak intervals marked by ‘*’ are clearly identical to the real seizure (ictal) intervals, indicating that the spectral method correctly detected the three GTCSs and resulted in no false-positive detection. However, low Stdev values were observed for the seizure intervals, as seen in Figure 5c. Note the high Stdev values at interictal epochs marked by “**”. Such a higher Stdev caused by normal motion can be falsely identified as a GTCS motion. Such problematic detection can increase the false-positive rate.

**Figure 6:**
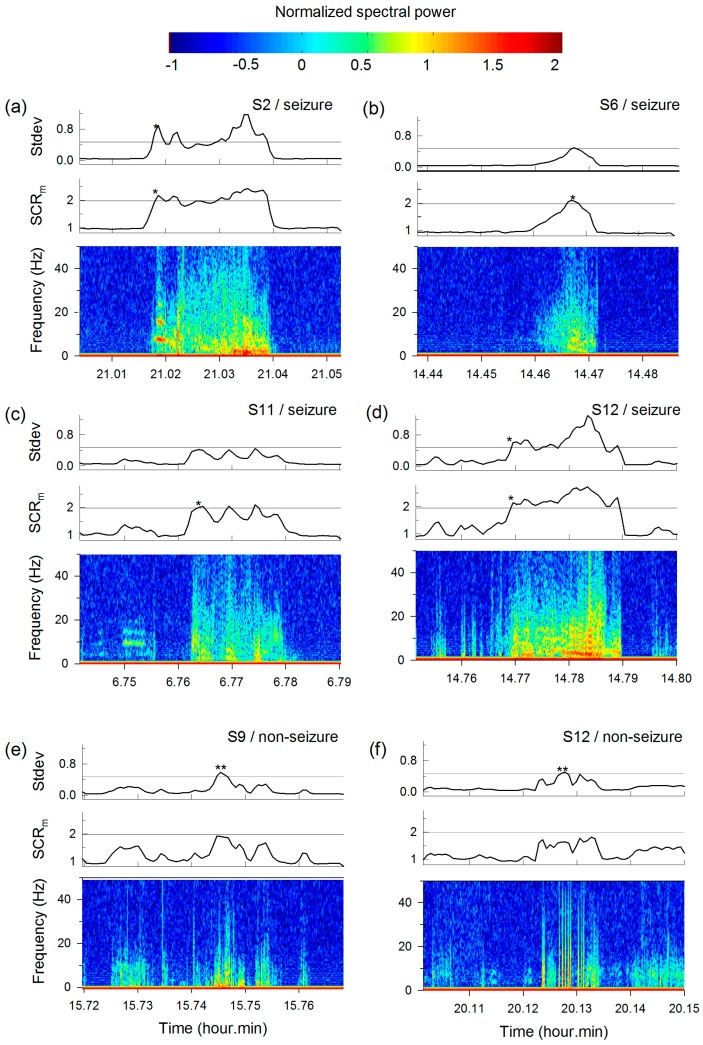
Examples of power spectra during either (**a**–**d**) seizure events or (**e**,**f**) normal movements. The SCR and Stdev of the ACM data are plotted as a function of recording time. ‘*’ and ‘**’ denote the true positives and false positives, respectively.

Figure 6 shows an example of the spectral information, SCR, and Stdev of either ictal (Figure 6a–d) or interictal (Figure 6e,f) intervals from five subjects. For all GTCSs in Figure 6a–d, higher spectral powers from 0 to 40 Hz and higher were observed. The ‘*’ in each panel denotes detection of GTCS via the spectral method (SCR) or temporal method (Stdev). Each threshold for the two methods was optimized in terms of detection accuracy. Figure 6c shows an example of the outperformance of the spectral method on the detection sensitivity. GTCS was correctly detected by the SCRs, whereas the temporal method classified GTCS as normal movement. Note also the several intervals of non-seizure cases in Figure 6e,f. The spectral plots show high spectral powers in the 0 to 40 Hz interval even though normal movements were involved. Such motions were incorrectly detected as GTCS using the temporal method (Stdev) and are denoted as ‘**’, whereas they were not identified by the spectral method.

### 3.2. Comparison of the Spectral and Temporal Analysis Methods

**Figure 7:**
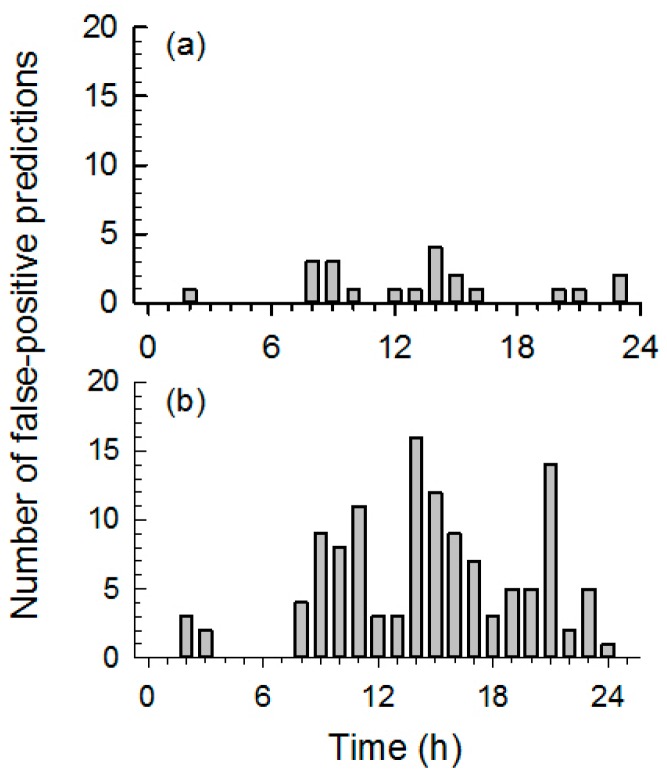
The incidence of false-positives during each 1-hr time interval for (**a**) the spectral analysis method and (**b**) the temporal analysis method. The horizontal axis represents the GTCS detection time from 00:00 to 24:00, and the bin width is set to 1 h.

The performance of the GTCS detection methods was evaluated using the 10-fold cross-validation method. To evaluate the performance of the spectral analysis, each fold-case was composed of nine training datasets used to compute the GDF[ω] and one test dataset to evaluate the detection performance. Both spectral and temporal methods optimized the thresholds from nine training datasets to detect all GTCS. Table 2 summarizes the sensitivity, specificity, PPV and FPR (cases/24 h) of the spectral analysis and temporal analysis methods. The sensitivity and FPR of the spectral analysis and temporal analysis methods were 100% and 2.0 cases/24 h and 90% and 11.8 cases/24 h, respectively. The spectral analysis method resulted in the better sensitivity and the lower false-positive rate than the temporal analysis method. Figure 7 shows the incidence of false-positive detections versus the detection time. Most false positive predictions occurred during the daytime in all cases. This suggests that the active normal movements during daytime may cause more false-positive predictions.

## 4. Discussion and Conclusions

This study developed and evaluated a new spectral analysis method using ACM data to detect GTCSs and reduce false-positive detections. The results showed that the spectral analysis method performed better to detect GTCS than the temporal analysis method did. The sensitivity and the FPR of the spectral analysis method were 100% and 2.0 cases/24 h, respectively, which are promising results. The temporal method produced a higher FPR by detecting non-seizure repetitive rhythmic movements during the daytime [5,6]. The spectral analysis method could reduce the FPR per day by 20% of that of the temporal analysis method.

Although video-EEG monitoring is a gold standard for seizure detection in clinical practice, seizure detection using the continuous analysis of scalp EEG is impractical for most patients with epilepsy and their caregivers in the home setting and during daily life [1,11,14]. The ACM recordings with an accurate seizure detection algorithm, as suggested in this study, can offer a less obtrusive alternative method. A reliable algorithm with a lower false positive detection rate is essential when it is used in real life. Repetitive rhythmic movements, such as brushing teeth, shaking a bottle or raising and lowering the arms, lead to false-positive detection [1]. Most of the spectral power of ACM patterns associated with epileptic seizures ranged from 4–8 Hz, whereas the spectral power of normal movement is generally concentrated below 0.8 Hz [10,17]. Therefore, the spectral method of GTCS detection using GTCSs template (Figure 4) can more effectively and efficiently detect the movements associated with epileptic seizures than the temporal method and exhibited a lower FPR. 

Table 3 presents a comparison of ACM-based seizure detection methods and their performance. Dalton et al. (2012) reported a dynamic time warping algorithm using temporal information such as standard deviation, mean, and zero-crossing. Milosevic et al. (2016) described support vector machine classification of temporal features (mean, standard deviation, and root mean square), spectral features (peak frequency and spectral energy), and temporal entropies. Various features and decision rules resulted in different sensitivities and FPRs. While most algorithms detected seizures with a sensitivity greater than 85%, the FPR varied from 2.0 to 11.8. Spectral information-based studies had better performance in terms of FPR. Further investigation of compromising these features and decision rules could help reduce false detections and improve sensitivity.

The present study has several concerns with regard to practical applications. First, this method is not sensitive enough to detect partial seizures without secondary generalization. However, while partial seizures are not without risk, most family members are more concerned with the detection of GTCS [1]. The multiple ACMs placed on the limbs and trunks of patients may make it more feasible to detect tonic, myoclonic, tonic-clonic, and partial seizures [19]. Second, if patients do not show hand movement, the events can be missed. The additional combined use of modalities such as electrocardiography or electromyography would increase the detectability of seizures and decrease false-positive alarms. Third, all the data in this study were recorded from inpatients during clinical epilepsy monitoring. Therefore, further large-scale studies are need. Incorporating a smartphone with the IMU to automatically call to alert a caregiver when a GTCS is detected and motion analysis using an accelerometer and gyroscope will be explored in future studies [20].

## Figures and Tables

**Table 1:** Subject, recording duration, and seizure data.

Subject	Age	Sex	Recording Time (h)	GTCS ID (Duration, s)
S1	28	F	25.87	-
S2	34	M	23.68	GTCS1 (90), GTCS2 (90)
S3	18	M	7.33	-
S4	39	M	8.41	-
S5	13	M	7.02	-
S6	50	F	6.95	GTCS3 (130), GTCS4 (256), GTCS5 (89)
S7	30	F	37.15	GTCS6(153)
S8	40	F	7.00	-
S9	33	M	55.83	-
S10	26	F	3.47	-
S11	52	M	38.12	GTCS7(165), GTCS8 (136), GTCS9 (108)
S12	33	M	25.75	GTCS10 (119)

**Table 2:** Seizure detection via spectral and temporal methods.

10-Fold Case	Spectral Analysis				Temporal Analysis			
	SEN	FPR	SPEC	PPV	SEN	FPR	SPEC	PPV
	(%)	(cases/24 h)	(%)	(%)	(%)	(cases/24 h)	(%)	(%)
1	100	0	100	100	100	0	100	100
2	100	0	100	100	100	0.9	99	50
3	100	0	100	100	100	1.9	99	33
4	100	0	100	100	100	0.9	99	50
5	100	1.9	99	33	100	9.7	99	9
6	100	2.9	99	25	100	21.3	99	4
7	100	6.8	99	12	100	30.1	99	3
8	100	5.8	99	14	0	16.6	99	0
9	100	0	100	100	100	16.6	99	5
10	100	2.9	99	25	100	20.4	99	4
Mean	100	2.0	99	61	90	11.8	99	26

SEN: sensitivity, FPR: false positive ratio, SPEC: specificity, PPV: positive predictive value.

**Table 3:** Comparison of ACM-based seizure detection methods.

	Spectral Analysis	Temporal Analysis	Milosevic et al. [18]	Dalton et al. [7]
ACM No.	1	1	2	4
Feature	SCR	Stdev.	Spectral, temporal	Temporal
Decision ^1^	Threshold	Threshold	SVM	DTW
No. of patients ^2^	5/12	5/12	7/56	5/5
No. of seizures	10	10	22	21
Seizure type ^3^	GTC	GTC	TC	C
Record duration	246 h	246 h	1998 h	130 h
Sensitivity (%)	100	90	86	91
FPR (case/24 h)	2.0	11.8	3.9	9.2

^1^ SVM: Support Vector Machine, DTW: Dynamic Time Warping; ^2^ Number of patients with seizures over total number of patients; ^3^ GTC: generalized tonic-clonic, TC: tonic-clonic, C: clonic seizures.
